# Supplementary material for: A small-molecule inhibitor of lectin-like oxidized LDL receptor-1 acts by stabilizing an inactive receptor tetramer state
Source: Commun Chem. 2020 Jun 10;3:75. doi: 10.1038/s42004-020-0321-2 (PMC9814544; doi:10.1038/s42004-020-0321-2)
Supplement: Supplementary file 1 — Supplementary Information [file 42004_2020_321_MOESM1_ESM.pdf]

Supplementary Information

A small-molecule inhibitor of lectin-like oxidized LDL receptor-1 acts by stabilizing an inactive receptor tetramer state

Author list

Gisela Schnapp[§], Heike Neubauer[§], Frank H. Büttner[§], Sandra Handschuh[§], Iain Lingard[*], Ralf Heilker[§], Klaus Klinder[§], Jürgen Prestle[§], Rainer Walter[§], Michael Wolff[§], Markus Zeeb[§], Francois Debaene[#], Herbert Nar[§] & Dennis Fiegen[§]

[§] Boehringer Ingelheim Pharma GmbH & Co. KG, 88397 Biberach, Germany

[*] Current address: Aptuit (Verona) Srl, an Evotec Company, Via Alessandro Fleming, 4, 37135 Verona, Italy

[#] NovAliX, BioParc, 850 bld Sebastien Brant, 67400 Illkirch, France

Corresponding author:

E-mail: dennis.fiegen@boehringer-ingelheim.com.
Phone: +49 7351 54-0.

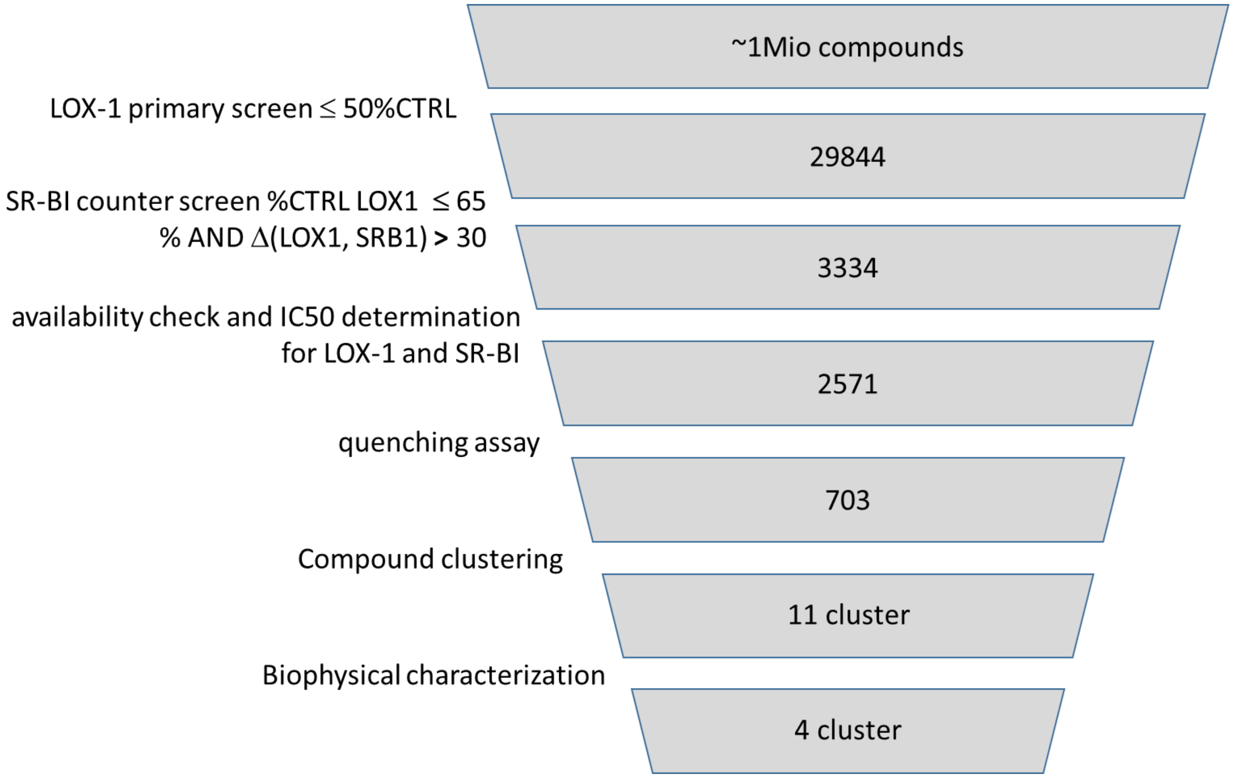

Supplementary Figure 1: The HTS screening cascade. The Boehringer Ingelheim screening library, around 1 million compounds, was tested in a primary screen. For confirmed hits IC50 curves have been determined. Compounds interfering with the assay have been weeded out by the quenching assay. Clustering of the remaining compounds identified 11 different classes. For four of these structural classes biophysical characterization could confirm CTLD target binding.

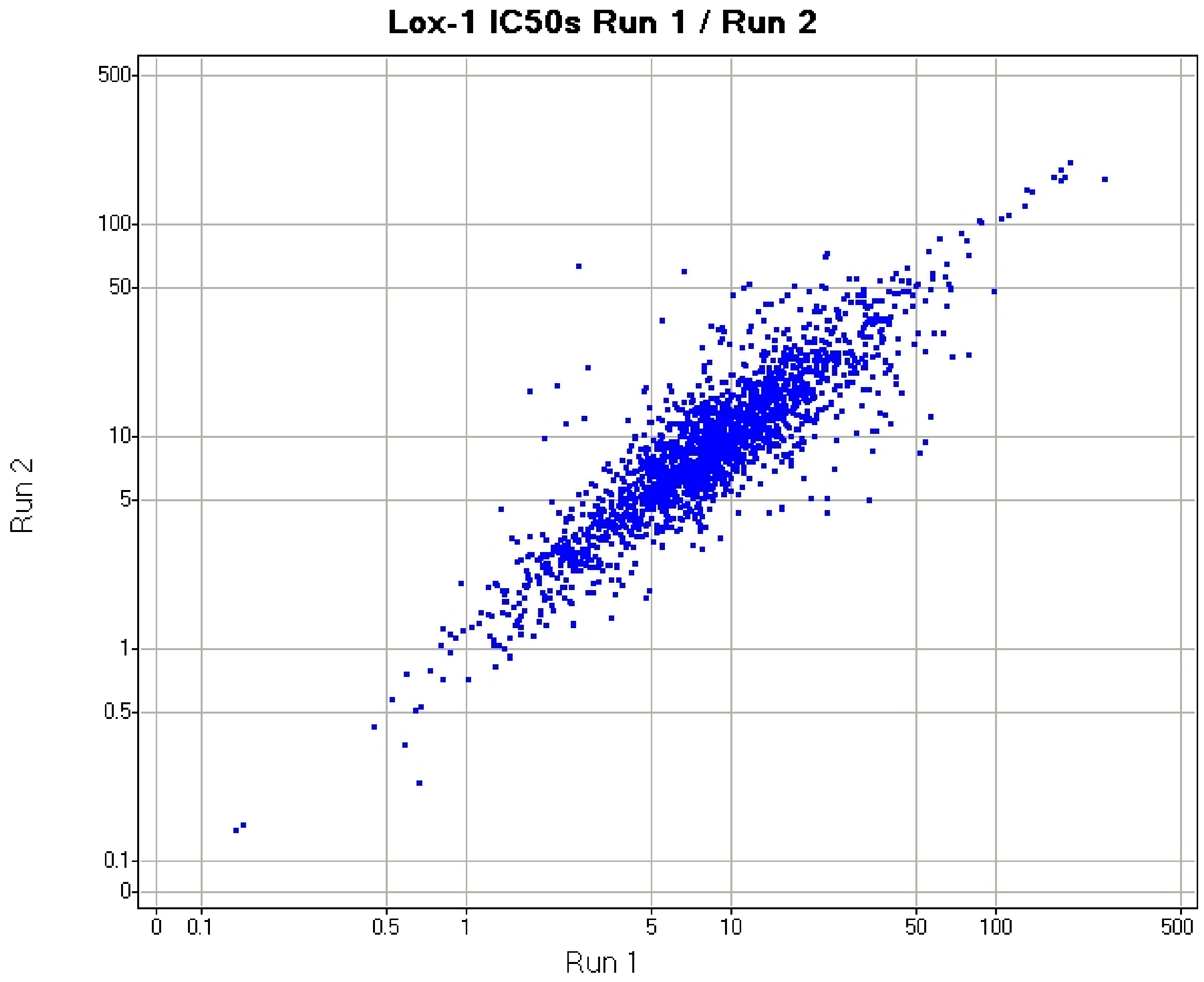

Supplementary Figure 2: 2571 compounds tested twice (Run1 and Run2) in the CHO-LOX-1 assay. The concentration on the x- and y-axis is given in µM.

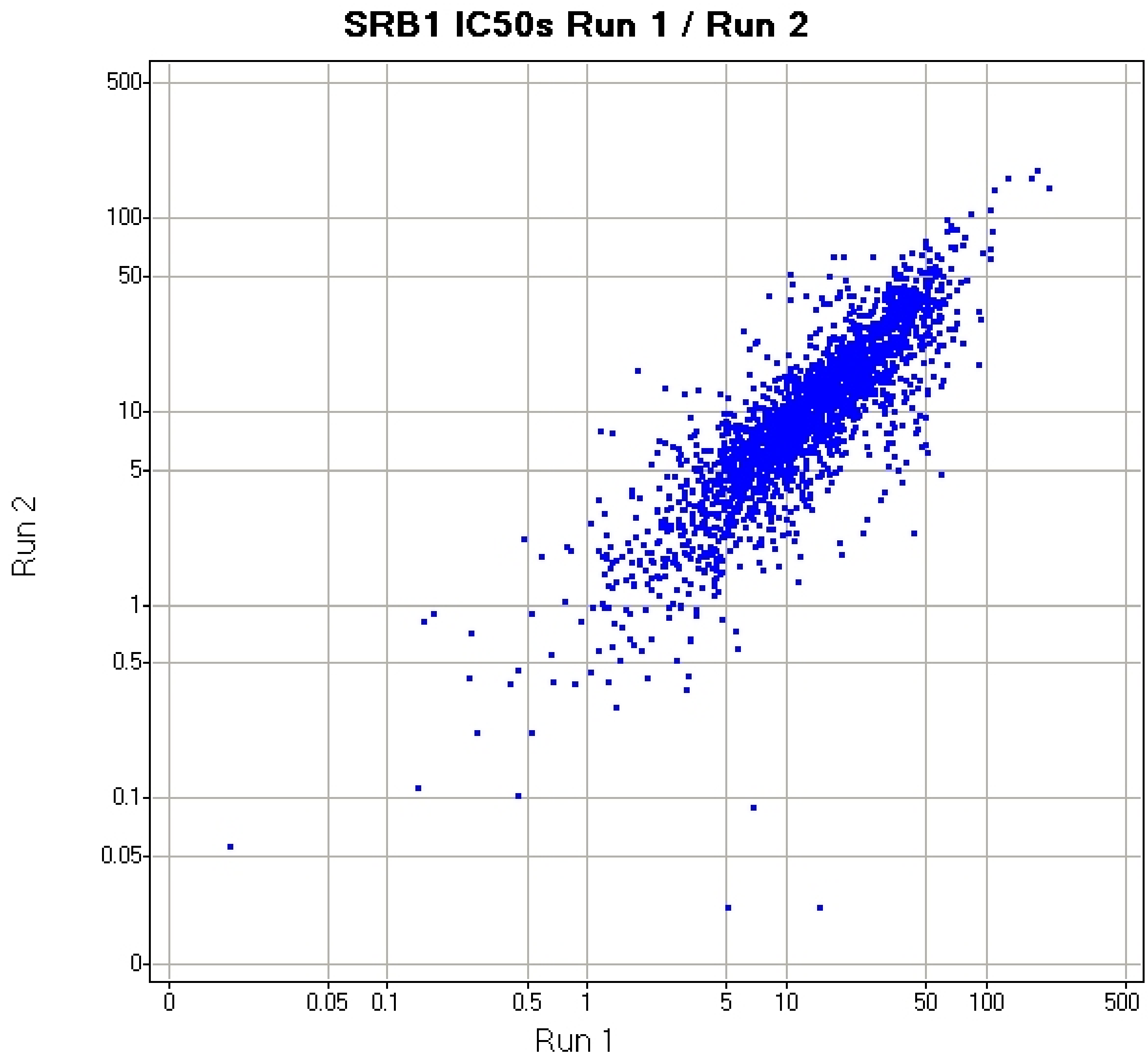

Supplementary Figure 3: 2571 compounds tested twice (Run1 and Run2) in the CHO-SR-B1 assay. The concentration on the x- and y-axis is given in µM.

Measurement	IC50 [µM]
1	7.55
2	7.15
3	3.34
4	2.01
5	5.85
6	6.95
7	5.11
8	4.97
Mean	5.37
Standard deviation	1.81

Supplementary Table 1: IC50 determinations of BI-0115 on LOX-1 from eight independent measurements.

Measurement	IC50 [µM]
1	88.80
2	>54.2
3	>100
4	>100
5	>100
6	>100
7	>172
8	>172

Supplementary Table 2: IC50 determinations of BI-0115 on SR-B1 from eight independent measurements.

Measurement	KD [µM]	Rmax theor [RU]"100%" Monomer	Rmax exp [RU]	Binding ratio
1	3.803	98.2	77.5	0.79
2	4.604	89.6	74.9	0.84
3	4.122	89.6	65.8	0.73
4	4.759	88.6	68.4	0.77
mean	4.322			0.78
standard deviation	0.381			0.04

Supplementary Table 3: SPR measurements of BI-0115 on LOX129.

Measurement	Temperature (°C)	[Syr] (µM)	[Cell] (µM)	Ligand in Cell	N (sites)	KD (µM)	ΔH (cal/mol)	ΔG (cal/mol)	-TΔS (cal/mol)
1	25	300	40	Yes	2.20	7.59	-1090	-6980	-5900
2	25	300	35	Yes	1.76	7.45	-1320	-7000	-5680
3	25	300	35	Yes	1.36	5.94	-3690	-7130	-3440
mean					***1.77***	***6.99***	***-2033***	***-7037***	***-5007***
standard deviation					***0.34***	***0.75***	***1175***	***66***	***1111***

Supplementary Table 4: ITC measurements of BI-0115 on LOX129.

	apo<br>129-273	apo<br>143-273	BI-0115<br>143-273
**Data collection**			
Wavelength [A]	0.9101	1.0000	1.0000
Space group	C 1 2 1	P2(1)2(1)2(1)	C 1 2 1
Cell dimensions			
a , *b* , *c* [Å]	76.8, 52.2, 109.8	40.5, 64.7, 101.9	150.8, 62.2, 116.3
α, β, γ [°]	90, 92, 90	90, 90, 90	90, 94, 90
Resolution [Å]	109.8 - 2.2 (2.27 - 2.16)	101.9 - 1.1 (1.17 - 1.11)	75.2 - 2.7 (2.743 - 2.734)
CC(1/2)	-	-	1.0 (0.9)
Rmerge [%]	2.9 (44.6)	5.0 (41.2)	6.6 (28.4)
<*I*/sd(*I*)>	19.2 (2.0)	18.9 (2.1)	12.5 (3.5)
Completeness [%]	97.0 (90.5)	88.8 (50.7)	97.1 (97.4)
Multiplicity	3.1	5.8	3.2
**Refinement**			
Resolution (Å)	55.0 - 2.2	34.3 - 1.1	39.0 - 2.7
No. reflections	22997	94306	27986
R_{work} / R_{free} (%)	21.0/23.3	13.2/16.2	22.2/24.6
No. Atoms			
Protein	3151	2376	8352
Ligand/ion	12	2*	86
Water	118	509	190
B -factors (Å^2)			
Protein	78.2	16.4	65.7
Ligand	74.7	15.3	36.9
Water	66.8	27.3	35.8
Estimated Coordinate Error (Å)	0.32	0.08 (ML)	0.44
R.m.s. deviations			
Bond lengths (Å)	0.008	0.011	0.006
Bond angles (°)	0.91	1.252	0.75

Supplementary Table 5: Data collection and refinement statistics of the non-liganded LOX129 and LOX143 structures and the LOX-1-BI-0115 complex. *The two Ni^{2+}-ions have been modeled based on geometry and distances[1]. The ions might stem from the affinity purification. Alternatively, two Zn^{2+}-ions could have been modeled, which are more abundant.

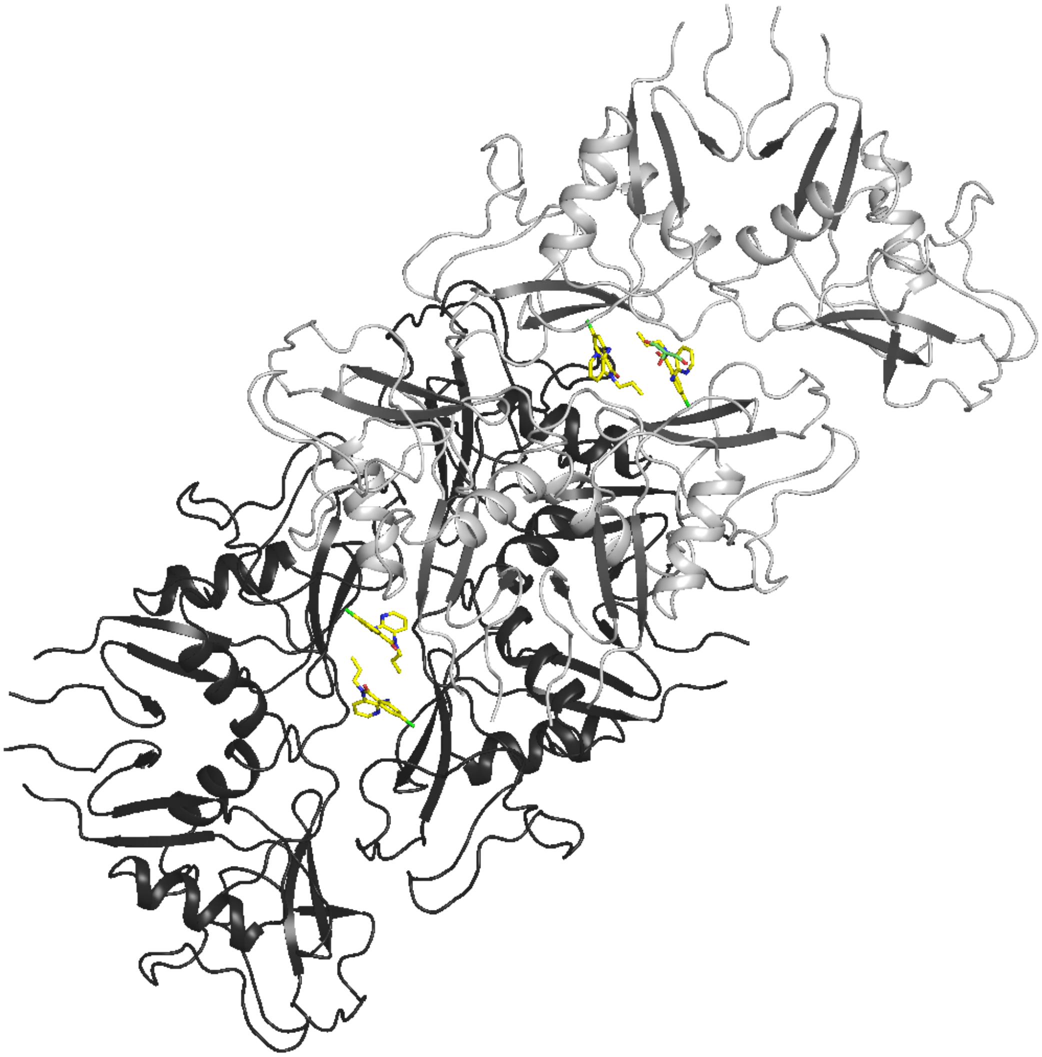

Supplementary Figure 4: Overview on the two tetramers of the asymmetric unit

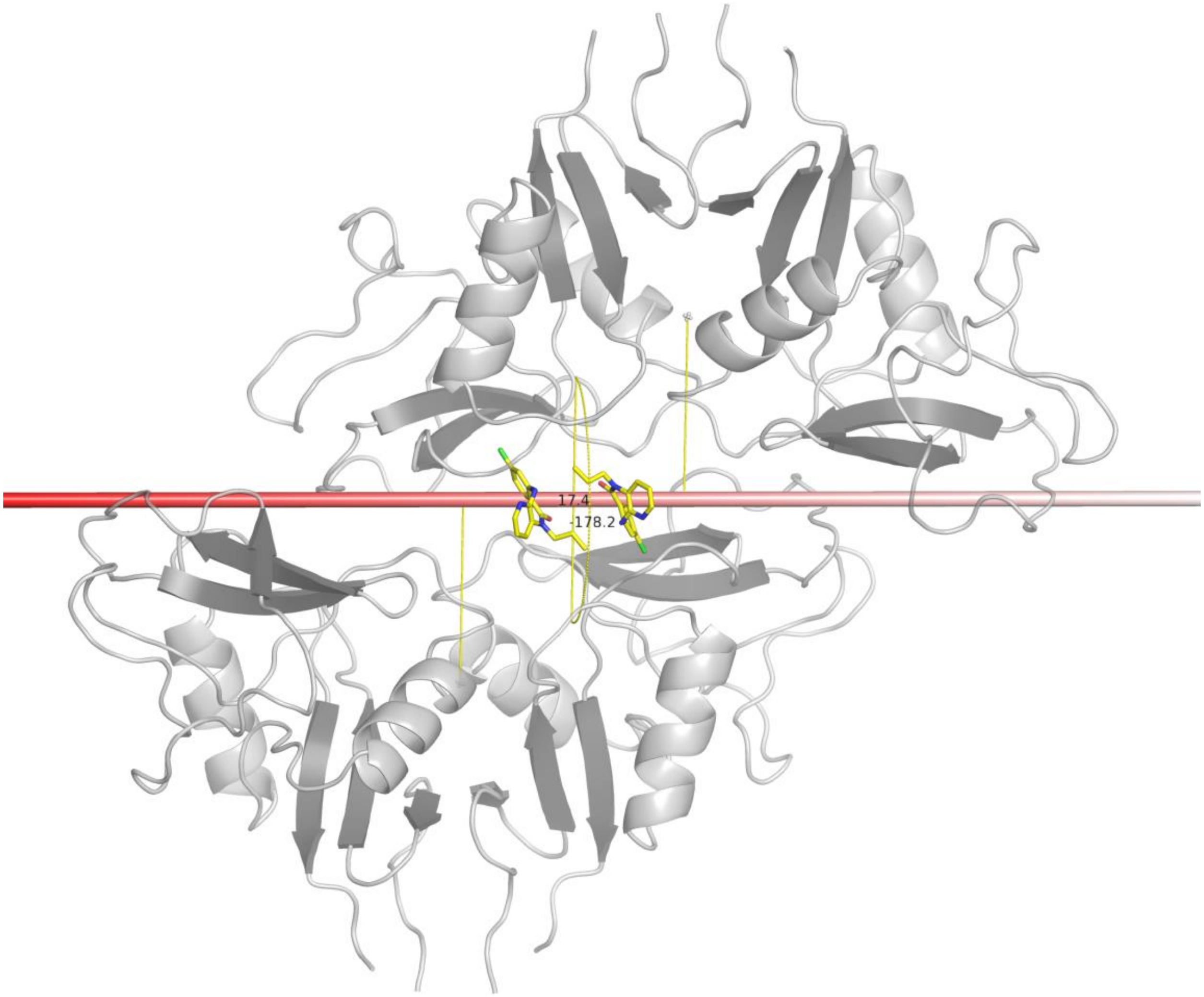

Supplementary Figure 5: Rotation and translation between the two dimers

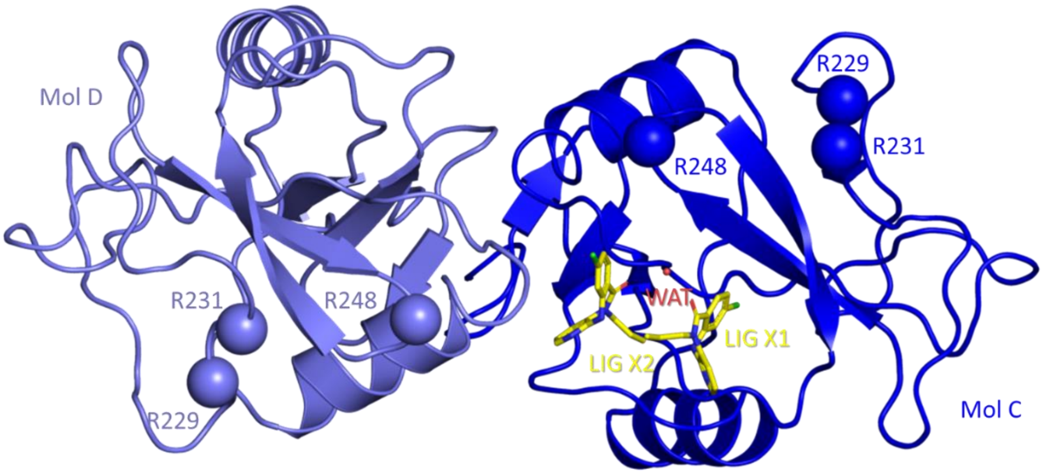

Supplementary Figure 6: BI-0115 binding site in relation to basic spine. Two molecules of BI-0115 are shown as yellow lines (LIG X1/LIG X2). C atoms are colored according to the LOX-1 molecule; O and N are in red and blue, respectively. LOX-1 molecules C are (Mol C) in blue and molecule D (Mol D) in light blue. The central water molecule (WAT) is indicated by a red filled circle. Position of the basic spine residues are highlighted by spheres and colored according to the LOX-1 molecule.

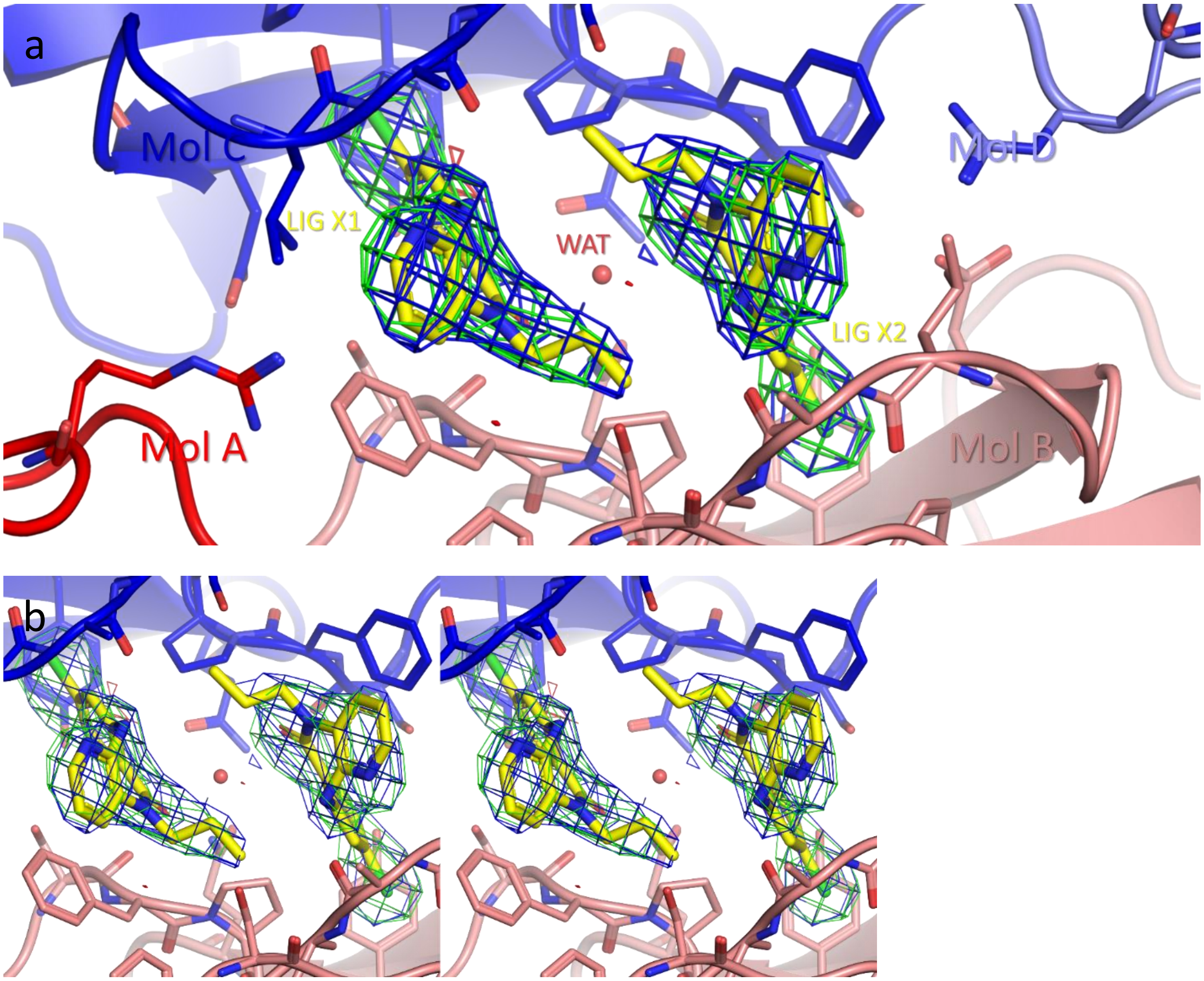

Supplementary Figure 7: a) 2fofc (blue, contoured at 1.0 sigma) and fofc (yellow, contoured at 3.0 sigma) electron densities surrounding BI-0115 (yellow) in the LOX-1 tetramer binding site. Densities have been calculated after refinement of the final model without ligands. b) Wall eye stereo picture of the same view as in a) and with the same contour levels for the electron densities.

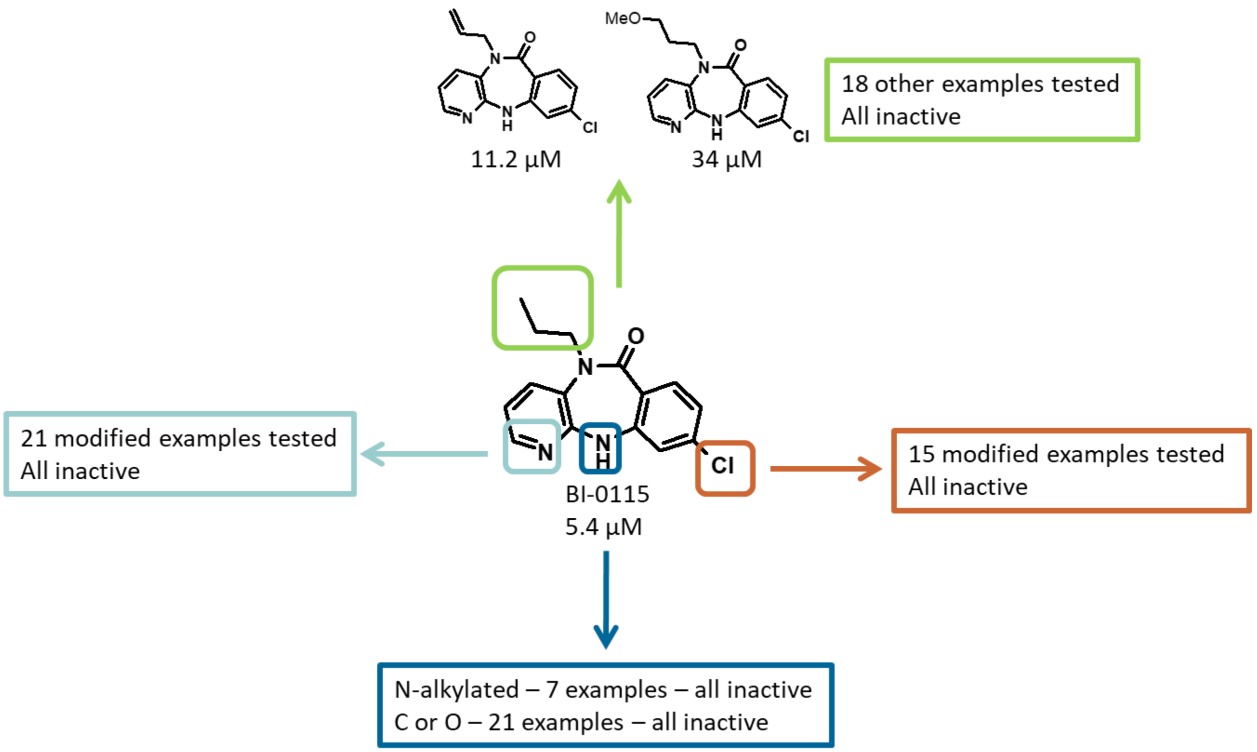

Supplementary Figure 8: Structure Activity Relationships around the BI-0115 compound series.

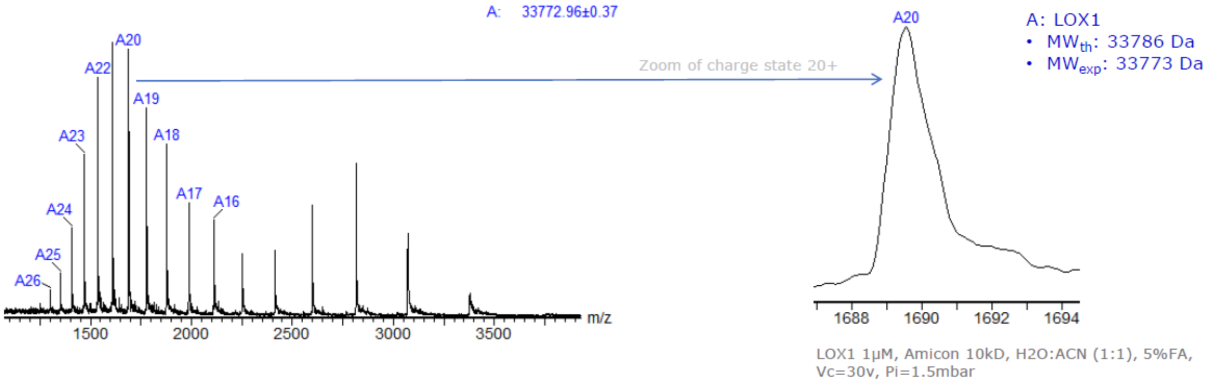

Supplementary Figure 9: Electrospray Ionization Mass Spectrometry data (ESI-MS) of LOX129 under denaturing conditions.

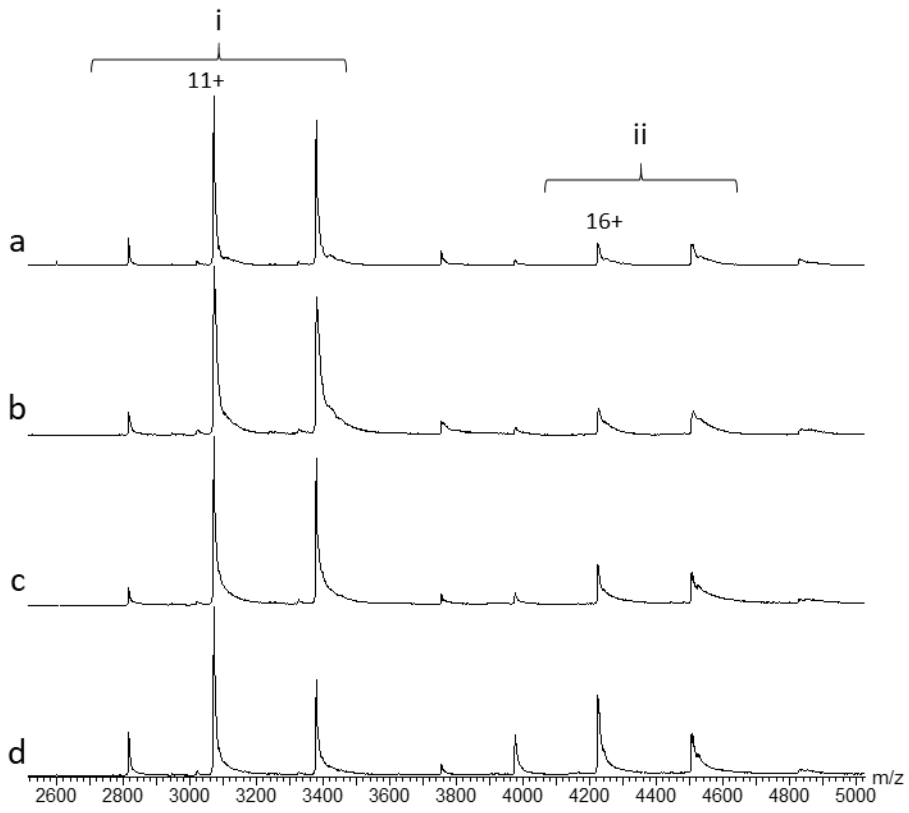

Supplementary Figure 10: Ligand induced LOX-1 tetramerisation at Vc = 60V. LOX-1 tetrameric state detected at minor form without ligand a) Increasing ligand concentration b) 2.5eq, c) 5eq, d) 10eq increase LOX-1 tetramerization species level. Ligand gas phase dissociation occurred at Vc = 60V: $(LOX\text{-}1)_2$ and $(LOX\text{-}1)_4$ measured without ligand. i: $(LOX\text{-}1)_2$ (MW: 33774 ±2Da), ii: $(LOX\text{-}1)_4$: 67550±4Da

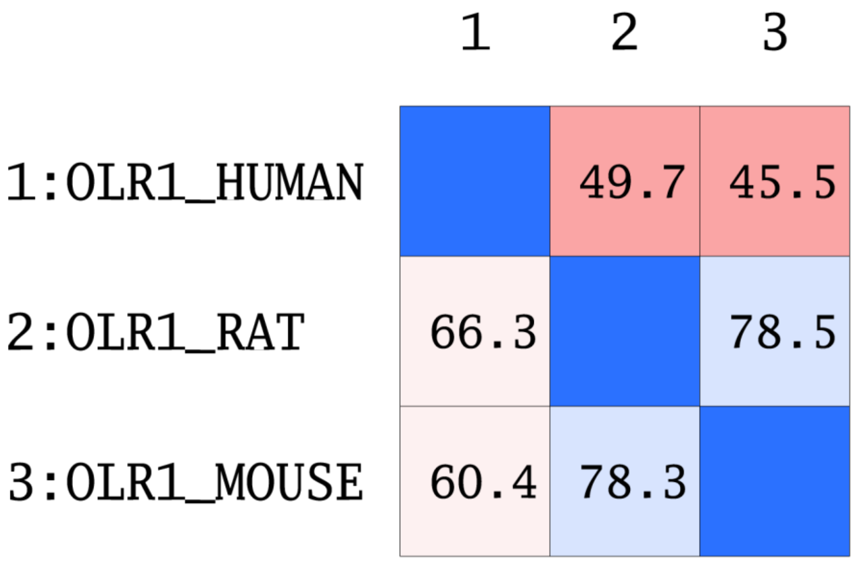

Supplementary Figure 11: Sequence conservation across human, rat and mouse. The given percent values reflect sequence identities for the full-length proteins.

```
OLR1_MOUSE  M T F D D . K M K P A N D E P D Q K S C G K K P K G L H L L   29
OLR1_RAT    M A F D D . K M K P V N G Q P D Q K S C G K K P K G L H L L   29
OLR1_HUMAN  M T F D D L K I Q T V K D Q P D E K S N G K K A K G L Q F L   30

S S P W W F P A A M T L V I L C L V L S V T L I V Q W T Q L R Q V S D L L K Q Y   69
S S T W W C P A A V T L A I L C L V L S V T L I V Q Q T Q L L Q V S D L L K Q Y   69
Y S P W W C L A A A T L G V L C L G L V V T I M V L G M Q L S Q V S D L L T Q E   70

Q A N L T Q Q D R I L E G Q M L A Q Q K A E N T S Q E S K K E L K G K I D T L T   109
Q A N L T Q Q D H I L E G Q M S A Q K K A E N A S Q E S K R E L K E Q I D T L T   109
Q A N L T H Q K K K L E G Q I S A R Q Q A E E A S Q E S E N E L K E M I E T L A   110

Q K L N E K S K E Q E E L L Q K N Q N L Q E A L Q R A A N S S E E S Q R E L K G   149
W K L N E K S K E Q E K L L Q Q N Q N L Q E A L Q R A V N A S E E S K W E L K E   149
R K L N E K S K E Q . . . . . . . . . . . . . . . . . . . . . . . . . . . . . .   120

K I D T I T R K L D E K S K E Q E E L L Q M I Q N L Q E A L Q R A A N S S E E S   189
Q I D I L N W K L N G I S K E Q K E L L Q Q N Q N L Q E A L Q K A E K Y S E E S   189
. . . . . . . . . . . . . . . . . . . . . . . . . . . . . . . . . . . . . . . .   120

Q R E L K G K I D T L T L K L N E K S K E Q E E L L Q K N Q N L Q E A L Q R A A   229
Q R E L K E Q I D T L S W K L N E K S K E Q E E L L Q Q N Q N L Q E A L Q R A A   229
. . . . . . . . . . . . . . . . . . . . . . M E L H H Q N L N L Q E T L K R V A   138

N F S G P C P Q D W L W H K E N C Y L F H . G P F S W E K N R Q T C Q S L G G Q   268
N S S G P C P Q D W I W H K E N C Y L F H . G P F N W E K S R E N C L S L D A Q   268
N C S A P C P Q D W I W H G E N C Y L F S S G S F N W E K S Q E K C L S L D A K   178

L L Q I N G A D D L T F I L Q A I S H T T S P F W I G L H R K K P G Q P W L W E   308
L L Q I S T T D D L N F V L Q A T S H S T S P F W M G L H R K N P N H P W L W E   308
L L K I N S T A D L D F I Q Q A I S Y S S F P F W M G L S R R N P S Y P W L W E   218

N G T P L N F Q F F K T R G V S L Q L Y S S G N C A Y L Q D G A V F A E N C I L   348
N G S P L S F Q F F R T R G V S L Q M Y S S G T C A Y I Q G G V V F A E N C I L   348
D G S P L M P H L F R V R G A V S Q T Y P S G T C A Y I Q R G A V Y A E N C I L   258

I A F S I C Q K K T N H L Q I .   363
T A F S I C Q K K A N L L L T Q   364
A A F S I C Q K K A N L R A Q .   273
```

Supplementary Figure 12: Sequence alignment of human, rat and mouse sequences with residues in 5-Angstrom distance around the BI-0115 binding site highlighted as red triangles. Identital residues are highlighted by a black background.

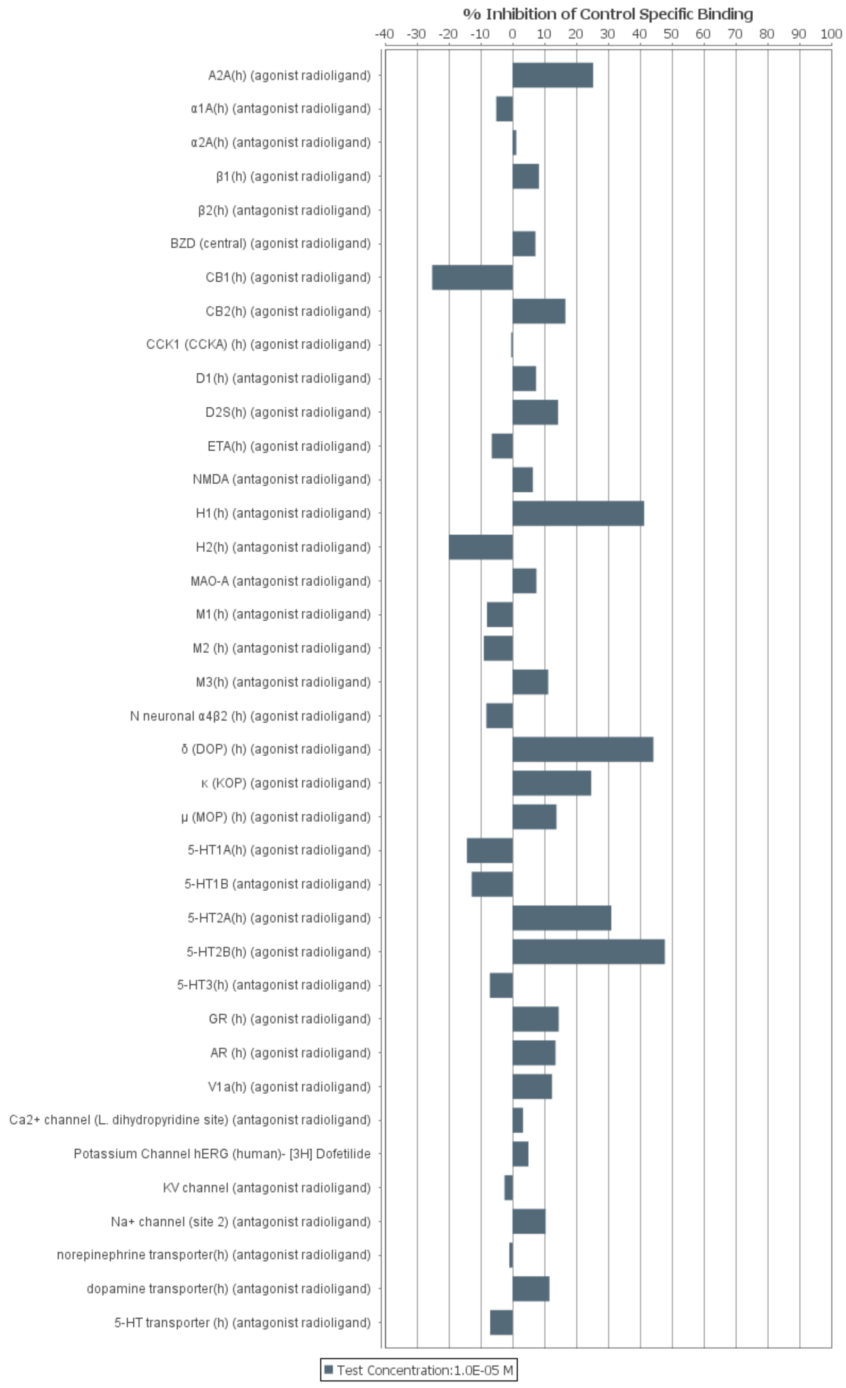

Supplementary Figure 13: Cerep panel for BI-0115 at a test concentration of 10 µM (n = 1)

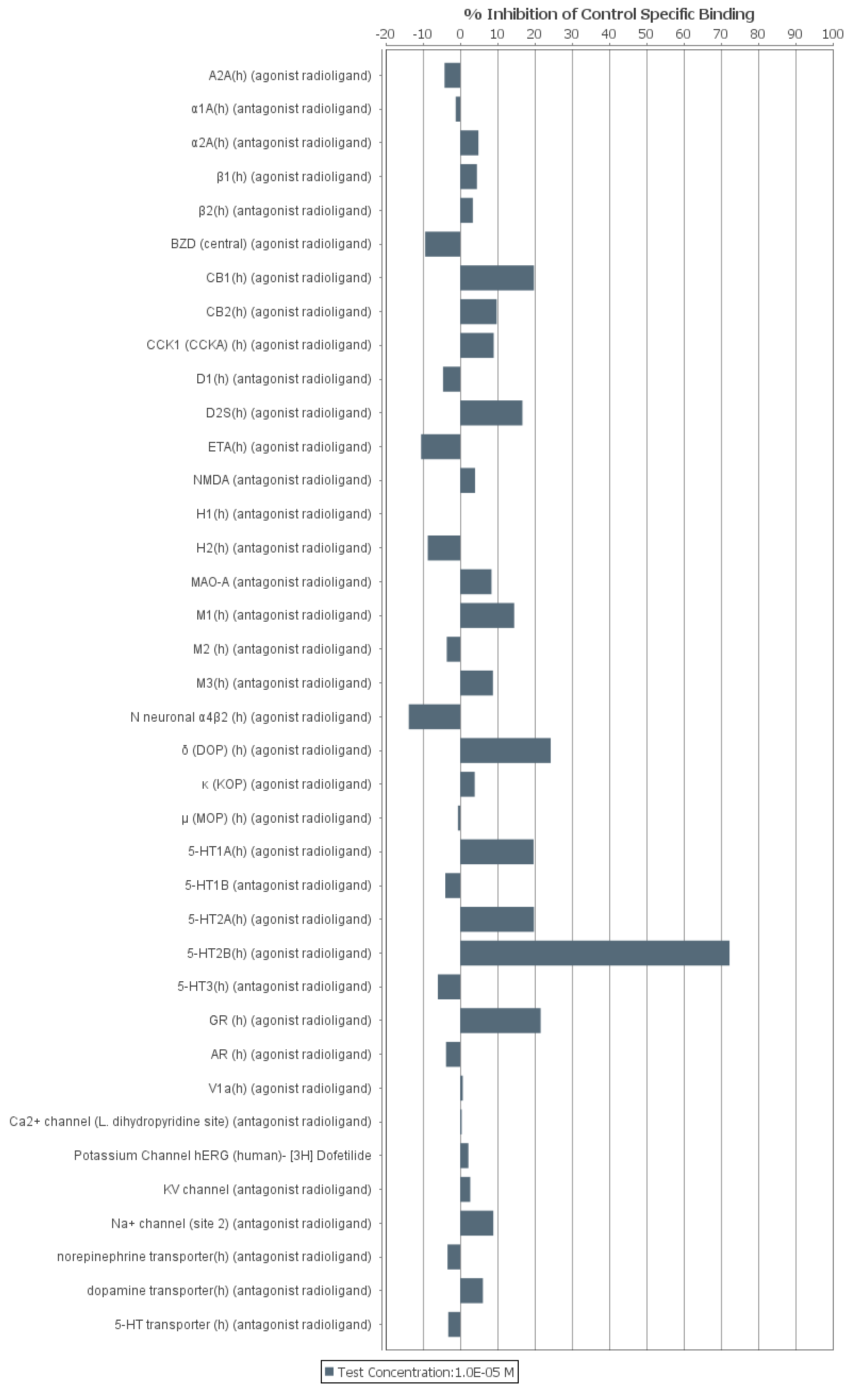

Supplementary Figure 14: Cerep panel for BI-1580 at a test concentration of 10 µM (n = 1)

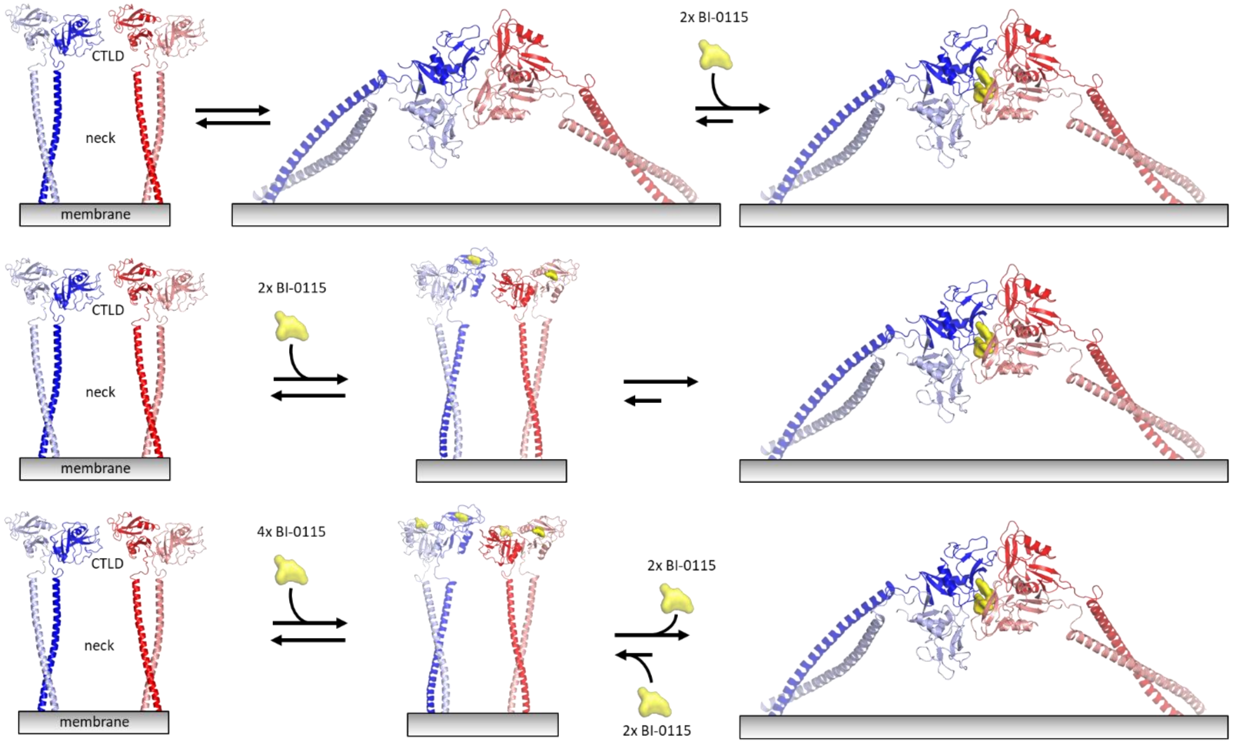

Supplementary Figure 15: Possible alternative intermediates on the reaction pathway to a 4:2 LOX-1:BI-0115 complex.

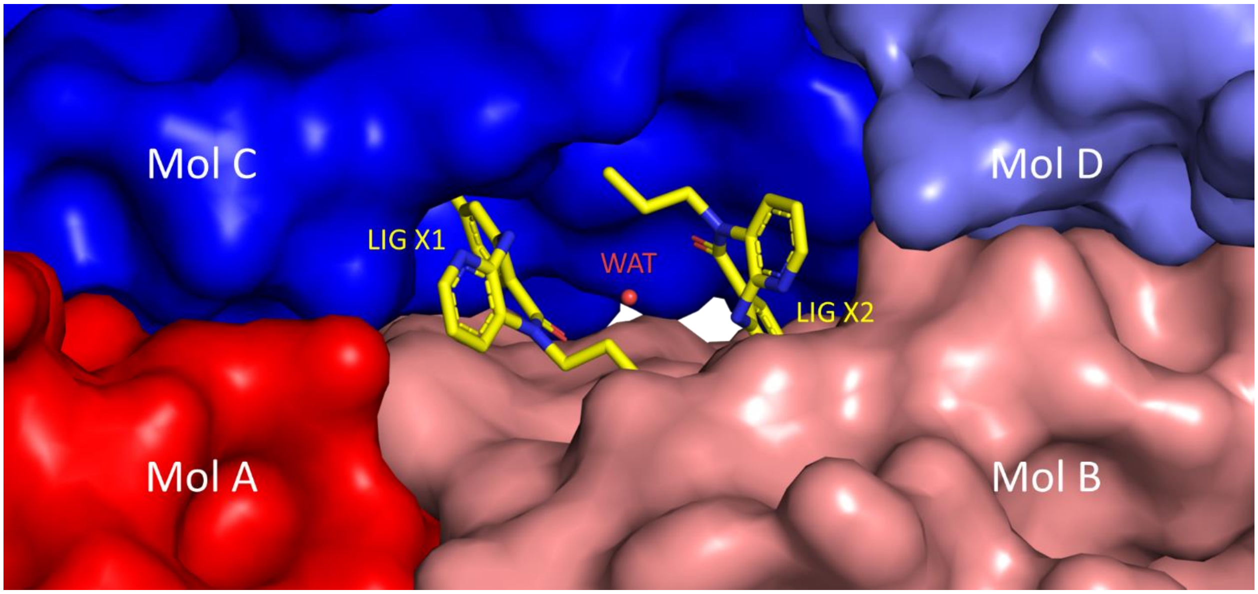

Supplementary Figure 16: Surface representation of the LOX-1 tetramer with two molecules of BI-0115 (C atoms in yellow and N atoms in blue) in line representation. The central water molecule (WAT) is indicated by a red sphere.

	1	2	3	4
1:OLR1_CTLD		39.1	31.2	32.4
2:CLC7A_CTLD	38.6		32.6	31.7
3:CL12B_CTLD	32.1	34.1		33.1
4:CLC9A_CTLD	32.1	31.9	31.9	

Supplementary Figure 17: Sequence conservation across human C-type lectin like domains of OLR1, CLC7A, CL12B and CLC9A. The given percent values reflect sequence identities for the CTLD domains.

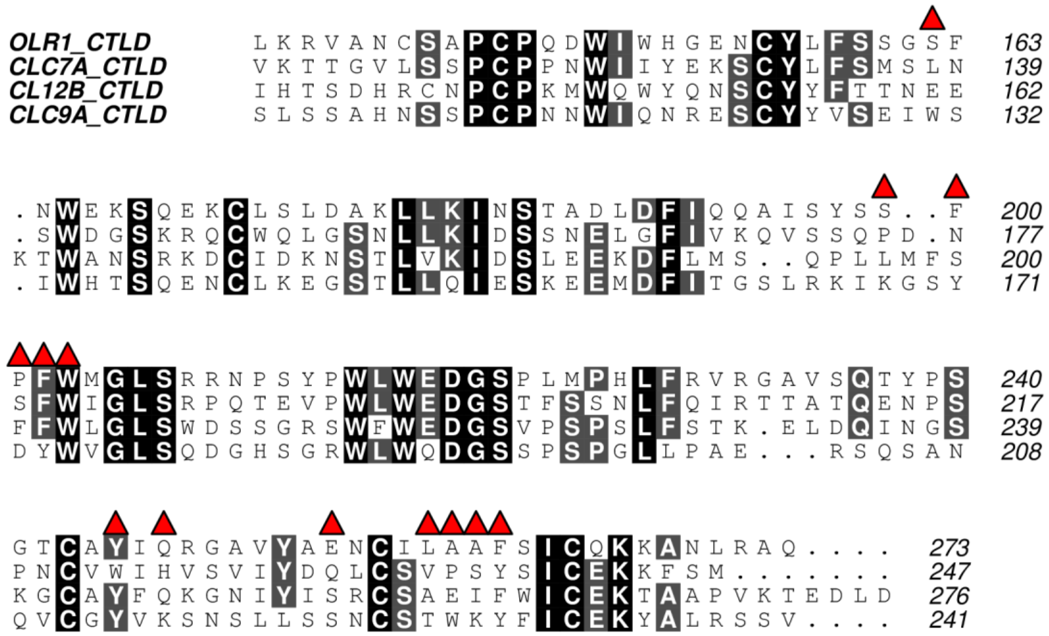

Supplementary Figure 18: Sequence alignment of human C-type lectin like domains of OLR1, CLC7A, CL12B and CLC9A with residues in 5-Angstrom distance around the BI-0115 binding site highlighted as red triangles.

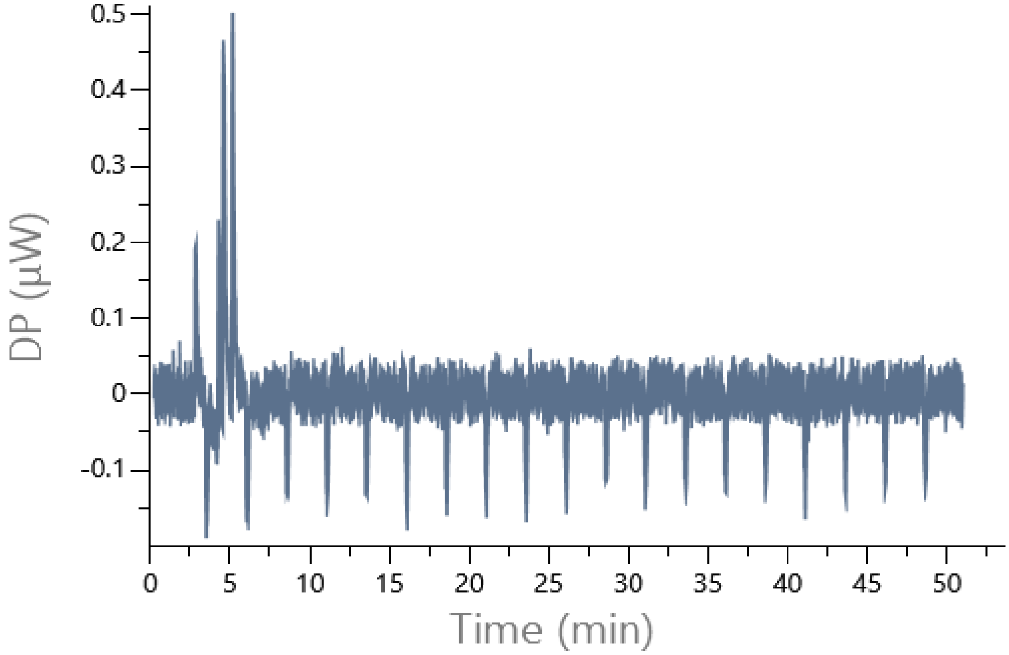

Supplementary Figure 19: ITC-Titration of 300 µM LOX129 dimer into buffer. The graph presents the raw data. No change of heat generation is indicated.

Supplementary methods

Compound synthesis

Experimental Part

The Examples that follow are intended to illustrate. The terms "ambient temperature" and "room temperature" are used interchangeably and designate a temperature of about 20 °C.

NMR experiments were recorded on Bruker Avance 400 MHz spectrometer at 303K. Sample BI-0115 was dissolved in 200µl DMSO-d6 and TMS was added as an internal standard and the sample BI-1580 was dissolved in 600µl DMSO-d6 and TMS was added as an internal standard. 1D-1H spectra were acquired with 30° pulse angle and a recycle delay of 2 sec with 64k number of points and a spectral width of 20.7 ppm. 1D-13C spectra were acquired with 90° pulse angle, proton broadband decoupling and a recycle delay of 4 sec, with 64k number of points and a spectral width of 276 ppm. Processing and analysis of spectra were performed with Bruker Topspin 3.2 software. The spectra were calibrated on TMS. Chemical shifts were reported in ppm on the δ scale.

HRMS data were recorded using a Waters Xevo G2 XS system with a Waters Acquity-UPLC. The mass calibration was performed using Lockspray (200 ng/mL LeuEnk; m/z 556.2771) ESI positive ion calibration.

TLC solvents

FM1: trichloromethane:CH:MeOH:NH_3	102:23:23:3
FM2: trichloromethane:EE	1:1
FM3: trichloromethane:CH:MeOH:NH_3	50:20:30:3

time (min)	Vol% water<br><br>(incl. 0.1% NH_4OH)	Vol% ACN	Flow [mL/min]
0.00	97	3	2.2
0.20	97	3	2.2
1.20	0	100	2.2
1.25	0	100	3
1.40	0	100	3

Supplementary Table 6: Analytical HPLC methods, Method A, Analytical column: XBridge C18 (Waters) 2.5 µm; 3.0 x 30 mm; column temperature: 60°C

Synthesis of Compound 1 (BI-1580)

Example I

N-(2-chloro-3-pyridinyl)-5-methyl-2-nitrobenzenecarboxamide

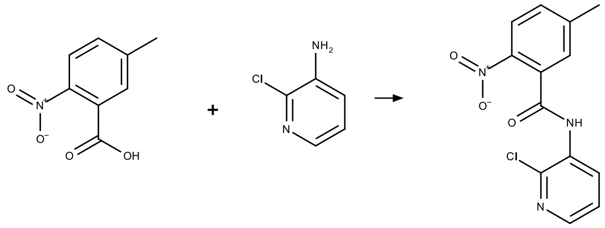

To 5-methyl-2-nitrobenzoic acid (181 g, 1.0 mol) in 300 ml HMPT is added thionylchlorid (72.5 ml, 1.0 mol) dropwise while strong stirring. The temperature is rising to 60°C. The mixture is stirred for 1 h at 60 - 40°C. Then 2-chloro-3-amino pyridine (130 g 1.0 mol) in 200 ml HMPT is added to the reaction. The temperature is rising to 80°C. The mixture is stirred for 1h at 80°C, quenched with ice-water and basified. The filtrate is filtered and washed with water. The residue is crystallized with acetonitrile to obtain 261 g 2-chloro-3-(2-nitro-5-methyl-benzoylamino)-pyridine as white crystals.

$C_{13}H_{10}ClN_3O_3$ (M = 291.7 g/mol)

R_f (TLC) FM1: 0.8

R_f (TLC) FM2: 0.65

MP: 153-155°C

Example II

2-Amino-N-(2-chloro-3-pyridinyl)-5-methyl-benzamide

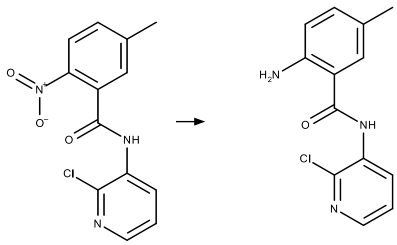

To 2-chloro-3-(2-nitro-5-methyl-benzoylamino)-pyridine (146 g, 0.5 mol) in 500 ml Dioxan is added Raney-nickel (20 g). The mixture is hydrogenated for 7 h at 70°C and 100at. DMF is added and warmed to solve the residue. The mixture is filtered and concentrated by evaporation. The residue is recrystallized in ACN. 116 g 2-amino-N-(2-chloro-3-pyridinyl)-5-methyl-benzamide is obtained.

$C_{13}H_{12}ClN_3O$ (M = 261.7 g/mol)

R_f (TLC) FM1: 0.9

R_f (TLC) FM2: 0.75

MP: 189-191°C

Example III

13-methyl-2,4,9-triazatricyclo[9.4.0.03,8]pentadeca-1(15),3,5,7,11,13-hexaen-10-one

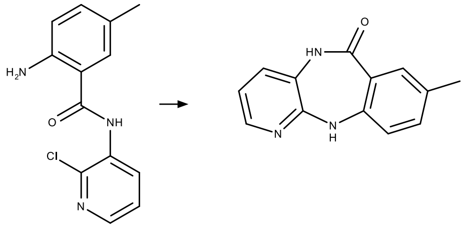

2-amino-N-(2-chloro-3-pyridinyl)-5-methyl-benzamide (115 g, 0.44 mol) in 230 ml sulfolan is heated. Exothermic reaction at 190°C. The mixture is stirred at 190°C for 30 min. The mixture is cooled to 80°C and 36 g sodium acetate in 360 mL water is added. The precipitation is filtered, washed with water and a small amount of methanol. The residue is recrystallized in diethylene glycol diethyl ether. 64.3 g 13-methyl-2,4,9-triazatricyclo[9.4.0.03,8]pentadeca-1(15),3,5,7,11,13-hexaen-10-one is obtained.

$C_{13}H_{11}N_3O$ (M = 225.3 g/mol)

R_f (TLC) FM1: 0.7

R_f (TLC) FM2: 0.5

MP: 256-258°C

Example IV

9-ethyl-13-methyl-2,4,9-triazatricyclo[9.4.0.03,8]pentadeca-1(11),3(8),4,6,12,14-hexaen-10-one

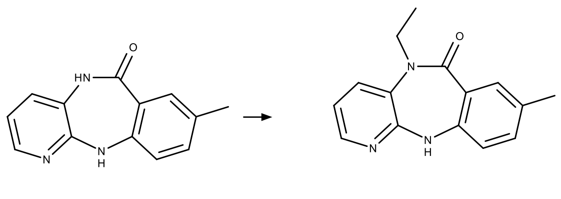

To 13-methyl-2,4,9-triazatricyclo[9.4.0.03,8]pentadeca-1(15),3,5,7,11,13-hexaen-10-one (170 g, 0.075 mol) in 150 ml DMF is added NaH (3.7 g, 0.085 mol). The reaction mixture is stirred at 50°C under inert atmosphere for 30 min. The reaction mixture is cooled and Iodoethane (14.0 g, 0.09 mol) is added dropwise. The reaction mixture is stirred at 120°C for 4h, hot filtered and the solvent is evaporated under reduced pressure. The residue is purified by silica gel column. Solvent: FM3

11.2 g 9-ethyl-13-methyl-2,4,9-triazatricyclo[9.4.0.03,8]pentadeca-1(11),3(8),4,6,12,14-hexaen-10-one is obtained.

$C_{15}H_{15}N_3O$ (M = 253.3 g/mol)

R_f (TLC) FM3: 0.6

MP: 182-188°C

Example V

9-ethyl-2,13-dimethyl-2,4,9-triazatricyclo[9.4.0.03,8]pentadeca-1(11),3,5,7,12,14-hexaen-10-one

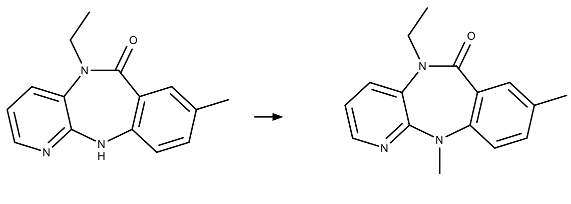

To 9-ethyl-13-methyl-2,4,9-triazatricyclo[9.4.0.03,8]pentadeca-1(11),3(8),4,6,12,14-hexaen-10-one (7.6 g, 0.03 mol) in 70 ml DMF is added NaH (1.9 g, 0.04 mol). The reaction mixture is stirred at 50°C under inert atmosphere for 30 min. A solution of Iodomethane (7.1 g, 0.05 mol) in 20 ml DMF is added dropwise to the reaction mixture. The reaction mixture is stirred at 100°C for 1h, hot filtered and the solvent is

evaporated under reduced pressure. The residue is diluted in chloroform and is extracted with water and sodium bicarbonate. The organic phase is evaporated under reduced pressure and is recrystallized with CH. 4.6 g 9-ethyl-13-methyl-2,4,9-triazatricyclo[9.4.0.03,8]pentadeca-1(11),3(8),4,6,12,14-hexaen-10-one is obtained.

mp: 127-129°C; TLC(FM2): R_f = 0.7; ^{1}H NMR (400 MHz, *DMSO-d_6*, 30°C) δ ppm 8.13 (dd, *J*=4.7, 1.6 Hz, 1 H) 7.81 (dd, *J*=8.0, 1.6 Hz, 1 H) 7.40 (d, *J*=2. Hz, 1 H) 7.26 (dd, *J*=8.4, 2.2 Hz, 1 H) 7.19 (dd, *J*=8.0, 4.7 Hz, 1 H) 7.07 (d, *J*=8.4 Hz, 1 H) 3.95 (m, 2 H) 3.25 (s, 3 H) 2.24 (s, 3 H) 1.15 (t, *J*=7.0 Hz, 3 H); ^{13}C NMR (101 MHz, *DMSO-d_6*, 30°C) δ ppm 167.1, 157.5, 149.0, 143.4, 132.7, 132.1, 131.6, 131.0, 129,4, 126.8, 119.8, 116.9, 44.3, 35.6, 19.8, 13.3; HRMS (m/z): $[M+H]^+$ calcd. for C16H18N3O, 268.1450; found, 268.1451

The purity of BI-1580 was >95 Mol%.

Synthesis of Compound 2 (BI-0115)

Example VI:

4-chloro-2-(4-methylbenzenesulfonamido)benzoic acid

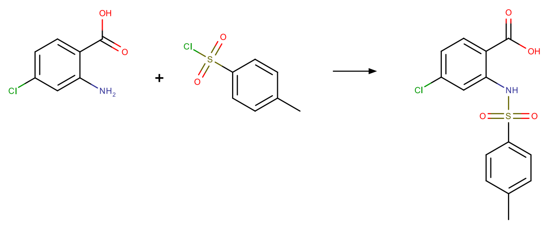

To a solution of sodium carbonate (130 g) in 500mL water is added 2-amino-4-chlorobenzoic acid (86 g) at 40°C. 4-methylbenzene-1-sulfonyl chloride is added and the mixture is stirred 3h at 85°C. Charcoal is added and the mixture is filtrated.

Then, 20% aq. HCl is added slowly, a solid appeared. 118g product is obtained by filtration.

$C_{14}H_{12}ClNO_4S$ (M = 325,0 g/mol)

MP: 190-194°C

Example VII:

4-chloro-2-(4-methylbenzenesulfonamido)benzoyl chloride

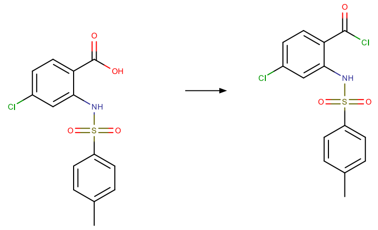

To 4-chloro-2-(4-methylbenzenesulfonamido)benzoic acid (118 g) in 600mL CCl_4 is added PCl_5 (96 g) at 75°C. The reaction mixture is refluxed for 1h. Charcoal is added to the solution, hot filtrated and the filtrate is cooled with iced water. The precipitation is filtered and washed with CCL_4 to obtain 68g product.

$C_{14}H_{11}Cl_2NO_3S$ (M = 343.0 g/mol)

MP: 147-150°C

Example VIII:

4-chloro-N-(2-chloropyridin-3-yl)-2-(4-methylbenzenesulfonamido)benzamide

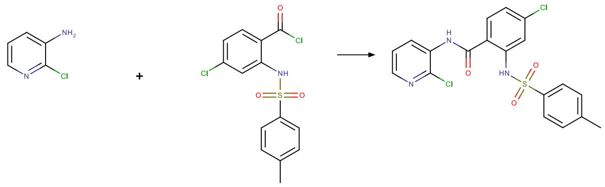

To 2-chloropyridin-3-amine (25.8 g) and N,N-dimethylaniline (25.7 g) in 440mL CCl4 is added 4-chloro-2-(4-methylbenzenesulfonamido)benzoyl chloride (68 g) portionwise at 75°C. The mixture is stirred 1h at 75°C. Then, 250mL 20% aq. HCl is added dropwise to the hot reaction mixture. The suspension is cooled down to 50°C, filtered and washed with water and ethanol to obtain 60g product.

$C_{19}H_{15}Cl_2N_3O_3S$ (M = 435.0 g/mol)

MP: 175-178°C

Example IX:

2-amino-4-chloro-N-(2-chloropyridin-3-yl)benzamide

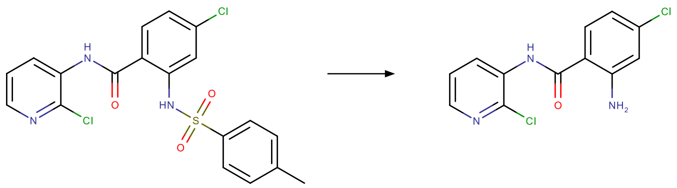

A mixture of 4-chloro-N-(2-chloropyridin-3-yl)-2-(4-methylbenzenesulfonamido)benzamide (357 g), 202mL conc. H2SO4 and 31mL water is stirred 2h at 100°C. The mixture is poured into ice and filtrated. The residue is diluted in water, basified with sodium hydroxide to pH 9-10. The precipitation is filtrated and washed with water to obtain 185g product.

$C_{12}H_9Cl_2N_3O$ (M = 281.0 g/mol)

MP: 199-201°C

Example X:

14-chloro-2,4,9-triazatricyclo[9.4.0.0^{3,8}]pentadeca-1(11),3,5,7,12,14-hexaen-10-one

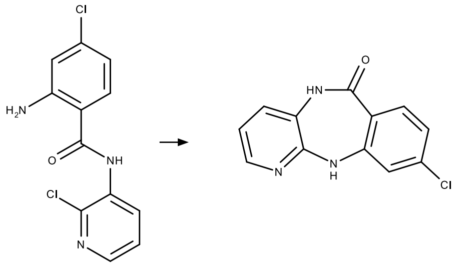

2-amino-4-chloro-N-(2-chloropyridin-3-yl)benzamide (14.1 g, 0.05 mol) in 56 ml sulfolan is added conc. sulfuric acid (0.3 ml) and the reaction mixture is heated at 130°C for 3h. The mixture is cooled to RT and 50 ml toluene is added. The precipitation is filtered, washed with a small amount of ethanol. The residue is diluted in water and basified with ammonia. The precipitate is filtered and washed with ethanol. 14-chloro-2,4,9-triazatricyclo[9.4.0.0^{3,8}]pentadeca-1(11),3,5,7,12,14-hexaen-10-one is obtained.

$C_{12}H_8ClN_3O$ (M = 245.6 g/mol)

MP: 210-215°C

Example XI:

14-chloro-9-propyl-2,4,9-triazatricyclo[9.4.0.0^{3,8}]pentadeca-1(11),3,5,7,12,14-hexaen-10-one

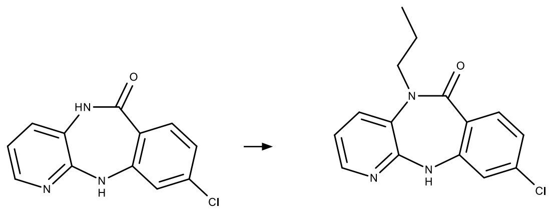

To 14-chloro-2,4,9-triazatricyclo[9.4.0.0^{3,8}]pentadeca-1(11),3,5,7,12,14-hexaen-10-one (7 g, 0.028 mol) in 70 ml DMF is added NaH (1.37 g, 0.031 mol). The reaction mixture is stirred at RT for 1.5h. 1-Iodopropane (4.8 g, 0.028 mol) is added dropwise. The reaction mixture is stirred at RT overnight. The reaction mixture is filtered and purified by HPLC (ACN/H_2O/NH_4OH). The fractions are combined, evaporated and dried at 50°C. 5.98 g 14-chloro-9-propyl-2,4,9-triazatricyclo[9.4.0.0^{3,8}]pentadeca-1(11),3,5,7,12,14-hexaen-10-one is obtained.

TLC (methode A): R_t = 0.99 min; ^{1}H NMR (400 MHz, *DMSO-d*$_6$, 30°C) δ ppm 8.71 (s, NH) 8.06 (dd, *J*=4.7, 1.5 Hz, 1 H) 7.82 (dd, *J*=8.0, 1.5 Hz, 1 H) 7.67 (d, *J*=8.5 Hz, 1 H) 7.31 (d, *J*=2.1 Hz, 1 H) 7.17 (dd, *J*=8.0, 4.7 Hz, 1 H) 7.05 (dd, *J*=8.5, 2.1 Hz, 1 H) 3.94 (t, *J*=7.0 Hz, 2 H) 1.45 (m, 2 H) 0.79 (t, *J*=7.4 Hz, 3 H); ^{13}C NMR (101 MHz, *DMSO-d*$_6$, 30°C) δ ppm 166.3, 155.1, 149.8, 144.0, 136.6, 133.9, 132.9, 127.0, 122.8, 121.7, 119.7, 118.6, 50.1, 20.3, 10.7; HRMS (m/z): $[M+H]^+$ calcd. for C15H15ClN3O, 288.0904; found, 288.0907

The purity of BI-0115 was >95 Mol%.

Abbreviations

ACN	acetonitrile
AcOH	acetic acid
Aq.	aqueous
°C	Degree celsius
CCl_4	Carbon tetrachloride
CH	cyclohexane
Conc.	concentrated
DCM	dichloro methane
DMF	*N,N*-dimethylformamide

DMSO	dimethyl sulfoxide
EE	ethyl acetate
EtOH	ethanol
Et_2O	diethyl ether
h	hour
H_2SO_4	Sulfuric acid
HCl	Hydrochlorid acid
HMPT	N,N,N',N',N'',N''-Hexamethylphosphorigtriamid
L	liter
MeOH	methanol
$NaHCO_3$	sodium bicarbonate
min	minute
mL	milliliter
NaH	Sodium hydride
NH_3	ammonia
PCl5	Phosphorus pentachloride
Ra-Ni	Raney Nickel
RT	room temperature (about 20°C)
sat.	saturated
TLC	Thin-layer chromatography on SiO2

Supplementary References

1. Zheng, H., Chruszcz, M., Lasota, P., Lebioda, L. & Minor, W. Data mining of metal ion environments present in protein structures. *J Inorg Biochem* **102**, 1765-76 (2008).
