# Supplementary material for: A small-molecule inhibitor of lectin-like oxidized LDL receptor-1 acts by stabilizing an inactive receptor tetramer state
Source: Commun Chem. 2020 Jun 10;3:75. doi: 10.1038/s42004-020-0321-2 (PMC9814544; doi:10.1038/s42004-020-0321-2)
Supplement: Supplementary file 3 — Reporting Summary [file 42004_2020_321_MOESM3_ESM.pdf]

nature research

Corresponding author(s): Dennis Fiegen

Last updated by author(s): May 13, 2020

Reporting Summary

Nature Research wishes to improve the reproducibility of the work that we publish. This form provides structure for consistency and transparency in reporting. For further information on Nature Research policies, see Authors & Referees and the Editorial Policy Checklist .

Statistics

For all statistical analyses, confirm that the following items are present in the figure legend, table legend, main text, or Methods section.

n/a	Confirmed	
☐	☒	The exact sample size (n) for each experimental group/condition, given as a discrete number and unit of measurement
☐	☒	A statement on whether measurements were taken from distinct samples or whether the same sample was measured repeatedly
☐	☒	The statistical test(s) used AND whether they are one- or two-sided<br>*Only common tests should be described solely by name; describe more complex techniques in the Methods section.*
☒	☐	A description of all covariates tested
☒	☐	A description of any assumptions or corrections, such as tests of normality and adjustment for multiple comparisons
☐	☒	A full description of the statistical parameters including central tendency (e.g. means) or other basic estimates (e.g. regression coefficient) AND variation (e.g. standard deviation) or associated estimates of uncertainty (e.g. confidence intervals)
☒	☐	For null hypothesis testing, the test statistic (e.g. F, t, r) with confidence intervals, effect sizes, degrees of freedom and P value noted<br>*Give P values as exact values whenever suitable.*
☒	☐	For Bayesian analysis, information on the choice of priors and Markov chain Monte Carlo settings
☒	☐	For hierarchical and complex designs, identification of the appropriate level for tests and full reporting of outcomes
☒	☐	Estimates of effect sizes (e.g. Cohen's d, Pearson's r), indicating how they were calculated

Our web collection on statistics for biologists contains articles on many of the points above.

Software and code

Policy information about availability of computer code

Data collection	HTS data:<br>Biolab (BI proprietary software)<br><br>Fluorescence microscopy:<br>IN Cell Analyzer 3000™ (General Electric [GE] Healthcare)<br><br>Biophysics:<br>MicroCal iTC200 software<br>Biacore T200 Evaluation Software 2.0<br><br>NMR:<br>Bruker Topspin 2.1<br><br>Native MS:<br>MassLynX 4.1
Data analysis	HTS data:<br>Spotfire<br>Biolab (BI proprietary software)<br><br>Dose response curves:<br>GraphPad Prism 8<br>Excel2016

Fluorescence microscopy:
IN Cell Analyzer 3000™ (General Electric [GE] Healthcare)

Biophysics:
MicroCal PEAQ-ITC Analysis Software
MicroCal iTC200 software
Biacore T200 Evaluation Software 2.0
GraphPad Prism 8
Excel2016

NMR:
Bruker Topspin 3.0

Native MS:
MassLynX 4.1

X-ray:
PyMOL(TM) 2.3.4
MOE 2019.0101
autoPROC Version: 1.1.6 <2015-12-14 01:12:12>, 0.5.2 <2009-07-17 09:48:30>, 0.5.5 <2009-12-01 09:27:36>
XDS version (March 1, 2015), (December 6, 2007)
AIMLESS (version 0.5.17)
CCP4 6.1/6.5.019
SCALA 3.3.9
POINTLESS 1.3.1
autoBUSTER Versions: 2.11.6 (2015-12-14); 2.11.7 (3-OCT-2019)
PHENIX (1.17.1_3660)
grade Version: 1.2.9 <July 24 2014>
MOGUL 1.7(RC5), CSD as536be, with quantum mechanics RM1
corina 3.46
MOL22TNT Version: 2.5.14
Omega Version: 2.5.1.4

For manuscripts utilizing custom algorithms or software that are central to the research but not yet described in published literature, software must be made available to editors/reviewers. We strongly encourage code deposition in a community repository (e.g. GitHub). See the Nature Research guidelines for submitting code & software for further information.

Data

Policy information about availability of data

All manuscripts must include a data availability statement. This statement should provide the following information, where applicable:
- Accession codes, unique identifiers, or web links for publicly available datasets
- A list of figures that have associated raw data
- A description of any restrictions on data availability

X-ray:
PDB: The crystallographic data for the LOX-1 extracellular domain in complex with BI-0115 is deposited under accession code 6TL9. The non-liganded LOX143 structure is deposited under accession code 6TL7. The non-liganded LOX129 structure is deposited under accession code 6TLA.
Raw data for 6TL9 can be provided.
Raw data for 6TL7 and 6TLA is not available.

HTS:
All relevant data has been provided. Additional data cannot be provided.

Fluorescence microscopy:
Additional data can be provided if necessary.

Dose response data:
Raw data can be provided if necessary.

NMR:
Raw data can be provided if necessary.

Native MS
Raw data can be provided if necessary.

Chemistry:
Additional analytics data can be provided.

List of figures with associated raw data:
Figure 1a
Figure 1b
Figure 1c
Figure 2a
Figure 2b (additional data in Supplement Table 3)
Figure 2c (additional data in Supplement Table 4)
Figure 3 (see X-ray data availability above)
Figure 4a-d
Figure 5
Table 1

Supplement Figure 1
Supplement Figure 2
Supplement Figure 3
Supplement Table 1
Supplement Table 2
Supplement Table 3
Supplement Table 4
Supplement Table 5
Supplement Figure 4
Supplement Figure 5
Supplement Figure 6
Supplement Figure 7
Supplement Figure 8
Supplement Figure 9
Supplement Figure 10
Supplement Figure 13
Supplement Figure 14
Supplement Figure 15
Supplement Figure 16
Supplement Figure 19

Field-specific reporting

Please select the one below that is the best fit for your research. If you are not sure, read the appropriate sections before making your selection.

☒ Life sciences ☐ Behavioural & social sciences ☐ Ecological, evolutionary & environmental sciences

For a reference copy of the document with all sections, see nature.com/documents/nr-reporting-summary-flat.pdf

Life sciences study design

All studies must disclose on these points even when the disclosure is negative.

Sample size

Not relevant.

Data exclusions

No data were excluded.

Replication

Fluorescence microscopy:
Figure 1a shows representative images of 16 experiments.

Figure 1b: HTS assay window
+ Tet - Alexa oxLDL: n = 6
- Tet + Alexa oxLDL: n = 4
+ Tet + Alexa oxLDL: n = 6
30 nM MAB-LOX, Hycult: n = 6
TTEST statictics are included in the manuscript.

Figure 1c: Dose response curve
n = 2

Figure 2a: STD NMR
n = 1

Figure 2b: SPR
One representative of n = 4.

Figure 2c: ITC
One representative of n = 3.

Figure 4 a-d: native MS
n = 1

Table 1:
BI-0115 data
LOX-1 IC50: n = 8
Quench Assay at 30 µM: n = 1
SR-B1 IC50: n = 8
ITC: n = 3
SPR: n = 4
STD-NMR: n = 1
ESI-MS: n = 1
Solubility pH4/pH7: n = 1/n = 1
micr. Stab hLM/rLM/mLM: n = 1/n = 1/n = 1
PAMPA permeability: n = 1
hERG IC50: n = 1
CEREP panel data has been measured once.

BI-1580 data
LOX-1 IC50: n = 2
Quench Assay at 30 µM: n = 1
SR-B1 IC50: n = 1
CEREP panel data has been measured once.

Randomization: Not relevant.

Blinding: Not relevant.

Reporting for specific materials, systems and methods

We require information from authors about some types of materials, experimental systems and methods used in many studies. Here, indicate whether each material, system or method listed is relevant to your study. If you are not sure if a list item applies to your research, read the appropriate section before selecting a response.

Materials & experimental systems

n/a	Involved in the study	
☐	☒	Antibodies
☐	☒	Eukaryotic cell lines
☐	☐	Palaeontology
☐	☐	Animals and other organisms
☐	☐	Human research participants
☐	☐	Clinical data

Methods

n/a	Involved in the study	
☐	☐	ChIP-seq
☐	☐	Flow cytometry
☐	☐	MRI-based neuroimaging

Antibodies

Antibodies used: anti-human-LOX-1 antibody (Trinova Biochem / Hycult # HM2138)

Validation: Validation within this publication is shown in Figure 1b.

https://www.hycultbiotech.com/hm2138bt-50ug
References:
1. Delneste, Y et al; Involvement of LOX-1 in dendritic cell-mediated antigen cross-presentation. Immunity 2002, 17: 353
2. Jeannin, P et al; Complexity and complementarity of outer membrane protein A recognition by cellular and humoral innate immunity receptors. Immunity 2005, 22: 551
3. Parlato, S et al; LOX-1 as a natural IFN-;-mediated signal for apoptotic cell uptake and antigen presentation in edndritic cells. Blood 2010, 115: 1554

Eukaryotic cell lines

Policy information about cell lines

Cell line source(s)	Cell line: CHO-K1 T-REx-hLOX-1<br>This cell line was generated using the background cell line T-REx™-CHO Cell Line purchased from (ThermoFisher formerly Invitrogen: https://www.thermofisher.com/order/catalog/product/R71807#/R71807)<br><br>Into this cell line, the LOX-1 expression plasmid pTREx-EXP30-hLox-1 was cloned. This plasmid was constructed using the Gateway Cloning system (ThermoFisher formerly Invitrogen): pT-Rex-DEST-30 into which the human hLOX-1 cDNA was cloned. This then resulted in the used cell line CHO-K1 T-REx-hLOX-1.
Authentication	No specific authentication was done with the line.
Mycoplasma contamination	Mycoplasma contamination was tested using MycoAlert (Lonza).
Commonly misidentified lines (See ICLAC register)	No commonly misidentified cell line was used.

Palaeontology

Specimen provenance	*Provide provenance information for specimens and describe permits that were obtained for the work (including the name of the issuing authority, the date of issue, and any identifying information).*
Specimen deposition	*Indicate where the specimens have been deposited to permit free access by other researchers.*
Dating methods	*If new dates are provided, describe how they were obtained (e.g. collection, storage, sample pretreatment and measurement), where they were obtained (i.e. lab name), the calibration program and the protocol for quality assurance OR state that no new dates are provided.*

☐ Tick this box to confirm that the raw and calibrated dates are available in the paper or in Supplementary Information.

Animals and other organisms

Policy information about studies involving animals; ARRIVE guidelines recommended for reporting animal research

Laboratory animals	*For laboratory animals, report species, strain, sex and age OR state that the study did not involve laboratory animals.*
Wild animals	*Provide details on animals observed in or captured in the field; report species, sex and age where possible. Describe how animals were caught and transported and what happened to captive animals after the study (if killed, explain why and describe method; if released, say where and when) OR state that the study did not involve wild animals.*
Field-collected samples	*For laboratory work with field-collected samples, describe all relevant parameters such as housing, maintenance, temperature, photoperiod and end-of-experiment protocol OR state that the study did not involve samples collected from the field.*
Ethics oversight	*Identify the organization(s) that approved or provided guidance on the study protocol, OR state that no ethical approval or guidance was required and explain why not.*

Note that full information on the approval of the study protocol must also be provided in the manuscript.

Human research participants

Policy information about studies involving human research participants

Population characteristics	*Describe the covariate-relevant population characteristics of the human research participants (e.g. age, gender, genotypic information, past and current diagnosis and treatment categories). If you filled out the behavioural & social sciences study design questions and have nothing to add here, write "See above."*
Recruitment	*Describe how participants were recruited. Outline any potential self-selection bias or other biases that may be present and how these are likely to impact results.*
Ethics oversight	*Identify the organization(s) that approved the study protocol.*

Note that full information on the approval of the study protocol must also be provided in the manuscript.

Clinical data

Policy information about clinical studies

All manuscripts should comply with the ICMJEguidelines for publication of clinical research and a completedCONSORT checklist must be included with all submissions.

Clinical trial registration	*Provide the trial registration number from ClinicalTrials.gov or an equivalent agency.*
Study protocol	*Note where the full trial protocol can be accessed OR if not available, explain why.*
Data collection	*Describe the settings and locales of data collection, noting the time periods of recruitment and data collection.*
Outcomes	*Describe how you pre-defined primary and secondary outcome measures and how you assessed these measures.*

ChIP-seq

Data deposition

☐ Confirm that both raw and final processed data have been deposited in a public database such as GEO.

☐ Confirm that you have deposited or provided access to graph files (e.g. BED files) for the called peaks.

Data access links<br>*May remain private before publication.*	*For "Initial submission" or "Revised version" documents, provide reviewer access links. For your "Final submission" document, provide a link to the deposited data.*
Files in database submission	*Provide a list of all files available in the database submission.*
Genome browser session<br>(e.g. UCSC)	*Provide a link to an anonymized genome browser session for "Initial submission" and "Revised version" documents only, to enable peer review. Write "no longer applicable" for "Final submission" documents.*

Methodology

Replicates	*Describe the experimental replicates, specifying number, type and replicate agreement.*
Sequencing depth	*Describe the sequencing depth for each experiment, providing the total number of reads, uniquely mapped reads, length of reads and whether they were paired- or single-end.*
Antibodies	*Describe the antibodies used for the ChIP-seq experiments; as applicable, provide supplier name, catalog number, clone name, and lot number.*
Peak calling parameters	*Specify the command line program and parameters used for read mapping and peak calling, including the ChIP, control and index files used.*
Data quality	*Describe the methods used to ensure data quality in full detail, including how many peaks are at FDR 5% and above 5-fold enrichment.*
Software	*Describe the software used to collect and analyze the ChIP-seq data. For custom code that has been deposited into a community repository, provide accession details.*

Flow Cytometry

Plots

Confirm that:

☐ The axis labels state the marker and fluorochrome used (e.g. CD4-FITC).

☐ The axis scales are clearly visible. Include numbers along axes only for bottom left plot of group (a 'group' is an analysis of identical markers).

☐ All plots are contour plots with outliers or pseudocolor plots.

☐ A numerical value for number of cells or percentage (with statistics) is provided.

Methodology

Sample preparation	*Describe the sample preparation, detailing the biological source of the cells and any tissue processing steps used.*
Instrument	*Identify the instrument used for data collection, specifying make and model number.*
Software	*Describe the software used to collect and analyze the flow cytometry data. For custom code that has been deposited into a community repository, provide accession details.*

Cell population abundance	*Describe the abundance of the relevant cell populations within post-sort fractions, providing details on the purity of the samples and how it was determined.*
Gating strategy	*Describe the gating strategy used for all relevant experiments, specifying the preliminary FSC/SSC gates of the starting cell population, indicating where boundaries between "positive" and "negative" staining cell populations are defined.*

☐ Tick this box to confirm that a figure exemplifying the gating strategy is provided in the Supplementary Information.

Magnetic resonance imaging

Experimental design

Design type	*Indicate task or resting state; event-related or block design.*
Design specifications	*Specify the number of blocks, trials or experimental units per session and/or subject, and specify the length of each trial or block (if trials are blocked) and interval between trials.*
Behavioral performance measures	*State number and/or type of variables recorded (e.g. correct button press, response time) and what statistics were used to establish that the subjects were performing the task as expected (e.g. mean, range, and/or standard deviation across subjects).*

Acquisition

Imaging type(s)	*Specify: functional, structural, diffusion, perfusion.*
Field strength	*Specify in Tesla*
Sequence & imaging parameters	*Specify the pulse sequence type (gradient echo, spin echo, etc.), imaging type (EPI, spiral, etc.), field of view, matrix size, slice thickness, orientation and TE/TR/flip angle.*
Area of acquisition	*State whether a whole brain scan was used OR define the area of acquisition, describing how the region was determined.*

Diffusion MRI ☐ Used ☐ Not used

Preprocessing

Preprocessing software	*Provide detail on software version and revision number and on specific parameters (model/functions, brain extraction, segmentation, smoothing kernel size, etc.).*
Normalization	*If data were normalized/standardized, describe the approach(es): specify linear or non-linear and define image types used for transformation OR indicate that data were not normalized and explain rationale for lack of normalization.*
Normalization template	*Describe the template used for normalization/transformation, specifying subject space or group standardized space (e.g. original Talairach, MNI305, ICBM152) OR indicate that the data were not normalized.*
Noise and artifact removal	*Describe your procedure(s) for artifact and structured noise removal, specifying motion parameters, tissue signals and physiological signals (heart rate, respiration).*
Volume censoring	*Define your software and/or method and criteria for volume censoring, and state the extent of such censoring.*

Statistical modeling & inference

Model type and settings	*Specify type (mass univariate, multivariate, RSA, predictive, etc.) and describe essential details of the model at the first and second levels (e.g. fixed, random or mixed effects; drift or auto-correlation).*
Effect(s) tested	*Define precise effect in terms of the task or stimulus conditions instead of psychological concepts and indicate whether ANOVA or factorial designs were used.*

Specify type of analysis: ☐ Whole brain ☐ ROI-based ☐ Both

Statistic type for inference (See Eklund et al. 2016)	*Specify voxel-wise or cluster-wise and report all relevant parameters for cluster-wise methods.*
Correction	*Describe the type of correction and how it is obtained for multiple comparisons (e.g. FWE, FDR, permutation or Monte Carlo).*

Models & analysis

n/a	Involved in the study
☐	☐ Functional and/or effective connectivity
☐	☐ Graph analysis
☐	☐ Multivariate modeling or predictive analysis

Functional and/or effective connectivity	*Report the measures of dependence used and the model details (e.g. Pearson correlation, partial correlation, mutual information).*
Graph analysis	*Report the dependent variable and connectivity measure, specifying weighted graph or binarized graph, subject- or group-level, and the global and/or node summaries used (e.g. clustering coefficient, efficiency, etc.).*
Multivariate modeling and predictive analysis	*Specify independent variables, features extraction and dimension reduction, model, training and evaluation metrics.*
